# A Self-Powered Optogenetic System for Implantable Blood Glucose Control

**DOI:** 10.34133/2022/9864734

**Published:** 2022-06-16

**Authors:** Zhuo Liu, Yang Zhou, Xuecheng Qu, Lingling Xu, Yang Zou, Yizhu Shan, Jiawei Shao, Chan Wang, Ying Liu, Jiangtao Xue, Dongjie Jiang, Yubo Fan, Zhou Li, Haifeng Ye

**Affiliations:** ^1^Key Laboratory for Biomechanics and Mechanobiology of Ministry of Education, Beijing Advanced Innovation Centre for Biomedical Engineering, School of Biological Science and Medical Engineering, School of Engineering Medicine, Beihang University, Beijing 100191, China; ^2^CAS Center for Excellence in Nanoscience Beijing Key Laboratory of Micro-Nano Energy and Sensor, Beijing Institute of Nanoenergy and Nanosystems, Chinese Academy of Sciences, Beijing 101400, China; ^3^Shanghai Frontiers Science Center of Genome Editing and Cell Therapy, Biomedical Synthetic Biology Research Center, Shanghai Key Laboratory of Regulatory Biology, Institute of Biomedical Sciences and School of Life Sciences, East China Normal University, Dongchuan Road 500, Shanghai 200241, China; ^4^School of Nanoscience and Technology, University of Chinese Academy of Sciences, Beijing 100049, China; ^5^Center of Nanoenergy Research, School of Physical Science and Technology, Guangxi University, Nanning 530004, China; ^6^Institute for Stem Cell and Regeneration, Chinese Academy of Sciences, Beijing 100101, China; ^7^Chongqing Key Laboratory of Precision Optics, Chongqing Institute of East China Normal University, Chongqing 401120, China

## Abstract

Diabetes treatment and rehabilitation are usually a lifetime process. Optogenetic engineered designer cell-therapy holds great promise in regulating blood glucose homeostasis. However, portable, sustainable, and long-term energy supplementation has previously presented a challenge for the use of optogenetic stimulation *in vivo*. Herein, we purpose a self-powered optogenetic system (SOS) for implantable blood glucose control. The SOS consists of a biocompatible far-red light (FRL) source, FRL-triggered transgene-expressing cells, a power management unit, and a flexible implantable piezoelectric nanogenerator (i-PENG) to supply long-term energy by converting biomechanical energy into electricity. Our results show that this system can harvest energy from body movement and power the FRL source, which then significantly enhanced production of a short variant of human glucagon-like peptide 1 (shGLP-1) *in vitro* and *in vivo*. Indeed, diabetic mice equipped with the SOS showed rapid restoration of blood glucose homeostasis, improved glucose, and insulin tolerance. Our results suggest that the SOS is sufficiently effective in self-powering the modulation of therapeutic outputs to control glucose homeostasis and, furthermore, present a new strategy for providing energy in optogenetic-based cell therapy.

## 1. Introduction

As a chronic disease, diabetes is an important contributor to cardiovascular and cerebrovascular diseases, death, amputation, blindness, and renal failure. The prevalence and incidence of diabetes have risen sharply. In 2019, diabetes affected at least 463 million people worldwide and resulted in approximately 1.5 million deaths, posing a serious threat to human health [1]. For diabetic patients, effective maintenance of blood glucose requires strict control and regulation of diet and lifelong injections of glucagon-like peptide-1 (GLP-1) analogs or insulin to control blood glucose [2, 3]. Nevertheless, the execution method of regularly injecting drugs over daily to weekly time periods is often inconvenient and of high cost to patients [4]. In addition, long-term repeated injections of insulin cause a localized immune response or fibrous tissue hyperplasia, leading to lipoatrophy or fat hypertrophy at the injection site [5]. Consequently, a responsive, controllable, and portable system should be considered as an alternative strategy to improve blood glucose control.

Currently, the emergence of the optogenetics employs both optical and genetic principles to control various cells' functions [6–8]. Various optogenetic systems have been developed for treating a number of diseases [9, 10], including diabetes [11, 12]. The glucose-lowering hormone insulin or GLP-1 was expressed in optogenetically engineered cells triggered by a particular wavelength of light to maintain glucose homeostasis in diabetic mice. Therefore, the optogenetic system can regulate blood glucose homeostasis using an implanted light source *in vivo* without periodic medication injections. While this novel technology may improve current treatment methods for diabetes, its application typically involves large-sized extracorporeal devices to power the light source, which may reduce patient compliance. As such, it is critical to establish a sustainable power source that satisfies the miniaturization and portability requirements for optogenetics to be used in implantable bioelectronics.

The human body contains abundant biomechanical energy, such as heart beating, joint movement, and breathing. Emerging nanogenerator technology [13–17] has opened a route for ambient mechanical energy to be converted into electricity/electrical signal using the multiple conversion mechanisms of piezoelectric [18–21] and triboelectric effects [22–25]. Advantages of using nanogenerators include low cost, lightweight, high energy conversion efficiency, and allowing for multiple design principles and a wide selection of materials [26–28]. In addition, flexible encapsulation strategies of nanogenerator can enable it to maintain good output performance in a humid environment or *in vivo* [29–34]. Therefore, nanogenerators with these exceptional capabilities have shown great potential in helping scientists utilize bioelectronics in energy harvesting [35–38] and sensing [39–41] required for a self-powered system [42–44]. We conceive that if an implantable nanogenerator can convert biomechanical energy into electricity for powering an optogenetic system's light source, then this technology may further promote the development of optogenetic systems, such as those that could be used to control blood glucose in a portable way that would improve patient compliance.

In this study, we report a self-powered optogenetic system (SOS) for implantable blood glucose control based on the implantable piezoelectric nanogenerator (i-PENG) with significantly increased electric output performance. The SOS harvests biomechanical energy from the body to provide power to turn on the light that ultimately activates optogenetic designer cells to produce therapeutic outputs. We demonstrated that the SOS can activate optogenetic designer cells to produce the secreted embryonic alkaline phosphatase (SEAP) *in vitro*. Moreover, we showcase that SOS-mediated shGLP-1 production was able to restore blood glucose levels in a diabetic mouse model. These results suggest that SOS has great potential in long-term optogenetic-based therapy.

## 2. Results

### 2.1. Design and Construction of the SOS

**Figure 1:**
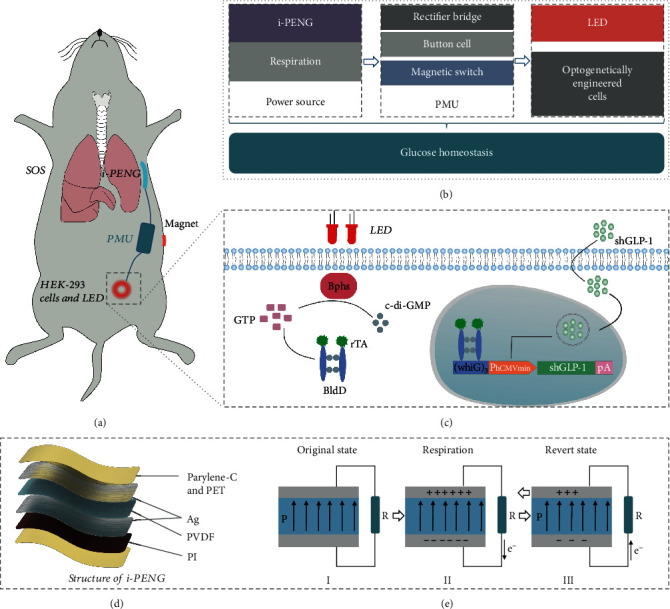
Overview of the design principle of the SOS. (a) Abstract diagram showing the SOS for regulating glucose homeostasis in diabetic mice. (b) Diagram showing the components of the SOS. The i-PENG converts biomechanical energy from murine respiration movement into electricity to provide energy to the power management unit that powers the LED. The PMU consists of three modules: a rectifier bridge, a button cell, and a magnetic switch. (c) Mechanism of FRL-inducible transgene expression in engineered human HEK-293 cells. (d) 3D structure of the i-PENG. The i-PENG consists of the poly (vinylidene fluoride) (PVDF) film, electrodes (Ag), and a substrate layer (polyimide, PI), which were encapsulated by biocompatible materials (polyethylene terephthalate (PET) and parylene-C). (e) The operating mechanism of the i-PENG.

The SOS is constructed by a flexible i-PENG, a power management unit (PMU), engineered HEK-293 cells, and an LED (Figure 1(a)). The biomechanical energy from murine respiration movements is harvested by the i-PENG for powering the LED light *via* the PMU, which then illuminates engineered HEK-293 cells. Figure 1(b) shows a diagram of the components in the SOS. In Figure 1(c), under far-red light (FRL) illumination from a LED, guanylate triphosphate (GTP) can be converted into cyclic diguanylate monophosphate (c-di-GMP) by bacterial photoreceptor BphS, which is specifically recognized by rTA-BldD, a fusion transactivator composed of BldD (*a Streptomyces coelicolor*-derived transcription factor), and a recombinant transcriptional activator (rTA). The increase in cytosolic c-di-GMP production triggers rTA-BldD dimerization and binding to the chimeric promoter *P*_FRL_ ((WhiG)_3_-*P*_hCMVmin_) that has been engineered to harbor BldD-specific operator DNA sites and eventually induces expression of human glucagon-like peptide 1 (shGLP-1). The i-PENG is an energy harvesting unit that consists of poly (vinylidene fluoride) (PVDF) film, electrodes (Ag), and a substrate layer (polyimide, PI), which were encapsulated by biocompatible materials (polyethylene terephthalate (PET) and parylene-C) (Figure 1(d)). Figure 1(e) shows the operating mechanism of the i-PENG. In this device, the positive piezoelectric effect of the PVDF film is used to harvests mechanical energy. External mechanical action (respiration) results in deformation of the PVDF film, which causes the internal electric dipole of the piezoelectric material to deflect, forming a potential difference between the two ends of the material. An external load circuit is connected the electrodes that alters to flow of electrons based on the potential difference. The periodic motion of respiration is loaded on the i-PENG, which can generate a continuous alternating current.

### 2.2. Characterization of the Implantable Piezoelectric Nanogenerator In Vitro

**Figure 2:**
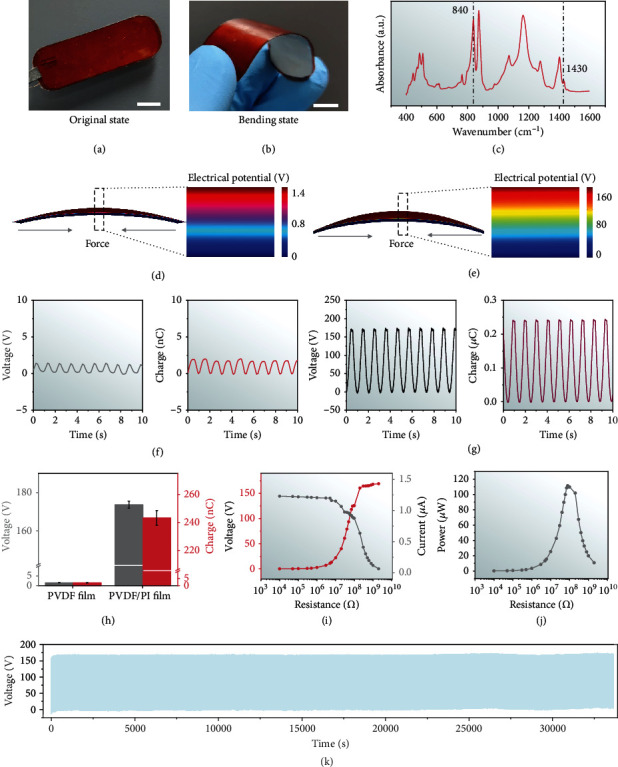
Characterization of the i-PENG. Photographs of the (a) original and (b) bent i-PENG demonstrating its good flexibility (scale bar = 1 cm). (c) Fourier transform infrared spectra of the PVDF film. Finite-element simulation of the voltage potential distribution for (d) the pure PVDF film and (e) the PVDF film integrated with the PI substrate. *V*_OC_ and *Q*_SC_ of (f) the pure PVDF film and (g) the PVDF film integrated with the PI substrate. These two different structure devices are driven by linear motors with a frequency of 1 Hz. (h) Statistical comparison of average *V*_OC_ and *Q*_SC_ of the pure PVDF film and the PVDF film integrated with the PI substrate. (i) *V*_OC_, *I*_SC_, and (j) peak power of the i-PENG at different load resistances. (k) Fatigue test of the i-PENG (~33,750 s, at 2 Hz).

While the i-PENG operates effectively in vivo, good flexibility is necessary in order to avoid damage to brittle tissues. Figures 2(a) and 2(b) display the mechanical elasticity of the device with an overall size of 2.2 cm × 6.2 cm × 400 *μ*m. The cross-sectional scanning electron microscope (SEM) image of i-PENG and the SEM images of nanostructure of the PVDF film are shown in Figure S1. The thickness was strictly controlled so that it would remain pliable and closely fit the tissue surface and respond to biomechanical motion with excellent sensitivity. The “bending degree” as an indicator is employed to define the flexibility of the i-PENG (Figure S2). The crystal structure of the PVDF film was investigated using the Fourier transform infrared (FTIR) method (Figure 2(c)). We identified the presence of characteristic absorption bands on the PVDF film at 840 and 1430 cm^−1^, which were associated with *β*-phase structure, indicating good piezoelectric properties. Efficient energy conversion by the i-PENG is critical for SOS function. The thin PI film was employed as a substrate layer, which can enhance the responsiveness of i-PENG to slight mechanical movements and thus improve the electrical output performance of the device. As shown in Figures 2(d) and 2(e), the *V*_OC_ (open-circuit voltage) of the pure PVDF film and the PVDF film with the substrate layer were simulated by finite element analysis, respectively. On the basis of not affecting the overall flexibility of the device, the construction of the substrate layer was found to significantly improve output performance. We also characterized the output performance of the device under laboratory conditions to verify the finite element simulation results. As shown in Figure 2(f), for the pure PVDF film, the *V*_OC_ and *Q*_SC_ (short-circuit transferred charge) were~1.4 V and~1.9 nC, respectively. In Figure 2(g), after the PVDF film was attached to the PI substrate, the *V*_OC_ and *Q*_SC_ reached ~170 V and~0.24 *μ*C. The electrical output performance was improved by nearly a hundred times (Figure 2(h)), and it was more suitable for the in vivo applications of converting biomechanical energy from respiratory movement into electricity. The mechanical durability of the i-PENG was also characterized. As shown in Figures 2(i) and 2(j), the *V*_OC_ and *I*_SC_ (short-circuit current) show opposite trends when the i-PENG was connected with different load resistances, the maximum output power reached 110 *μ*W, and the internal resistance of the i-PENG was about 100 M*Ω*. The device was connected to a button cell through a rectifier. The button cell could be charged from 2.0 V to 2.4 V in 24 h and discharged by LED load from 2.4 V to 1.3 V over 2 h (Figure S3). The voltage of the i-PENG maintained stable in compared with its initial state after ~67,500 working cycles (Figure 2(k) and Figure S4). The results show that the device has the characteristics of good robustness and excellent electrical output, which enables for long-term harvesting biomechanical energy *in vivo*.

### 2.3. Performance of the i-PENG In Vivo

**Figure 3:**
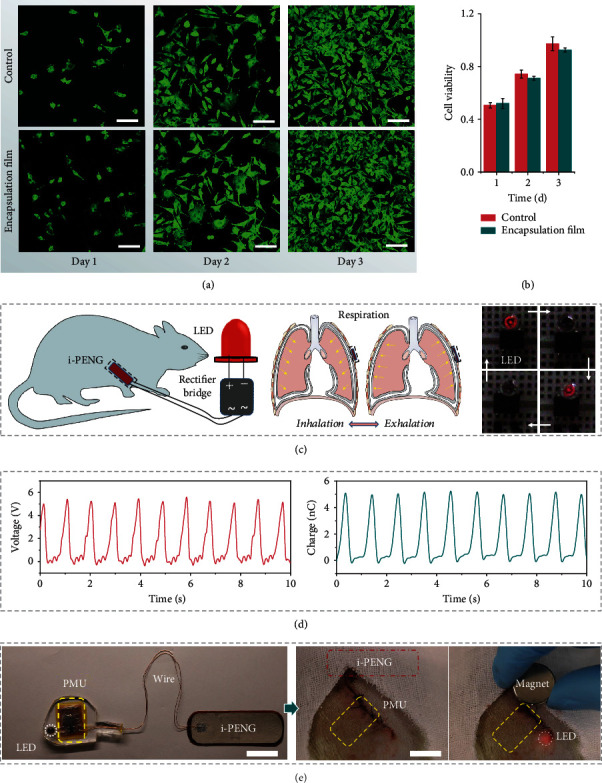
The performance of the i-PENG in vivo. (a) Fluorescence images (scale bar = 100 *μ*m) and (b) cell viability of stained fibroblasts after being cultured with encapsulation film from the i-PENG for 1, 2, and 3 days. (c) Schematic diagram of the i-PENG that converts the biomechanical energy from respiration into electricity for directly powering the LED. The blinking frequency of the LED is the same as the breathing rate of a SD rat. (d) Electrical output of the i-PENG *in vivo*. The *V*_OC_ and *Q*_SC_ of i-PENG in the SD rat were measured under anesthesia. (e) Photographs of the i-PENG-based SOS *in vivo* and controlled by a magnet (scale bar = 2 cm).

Precise biocompatibility for implantable devices is critical to avoid causing inflammation in the surrounding tissue. To evaluate the performance of the i-PENG *in vivo*, we first demonstrated the biocompatible of the i-PENG. Fibroblast cell line L929 was cultured on the encapsulation materials in a cell culture dish. The cytoskeletal structures were detected by fluorescence staining, suggesting that the cells in the control group and the experimental group were healthy (Figure 3(a)). As shown in Figure 3(b), the results of a CCK-8 assay showed that the cell viability in the experiment group was similar to that of control group after 3 days of culture. These data suggest that the encapsulation materials have appropriate biocompatibility and are nontoxic. To study the i-PENG-mediated energy conversion efficiency *in vivo*, the device was implanted into the subcutaneous region of the chest in SD (Sprague Dawley) rats (Figure 3(c)). The *V*_OC_ of this device could reach up to ~5 V, and the corresponding *Q*_SC_ was ~5 nC (Figure 3(d)). Due to the weak intensity of the breathing movement of the small animal, the *in vivo* output performance of the i-PENG was attenuated, but it could still provide enough energy to light up the LED (Supplementary Movie 1). These results show that the i-PENG has good energy conversion efficiency and motion sensitivity in rats, and we expect the SOS based on the i-PENG to successfully treat disease when coupled with optogenetic technology *in vivo* (Figure 3(e) and Supplementary Movie 2, 3).

### 2.4. SOS-Mediated Transgene Expression In Vitro

**Figure 4:**
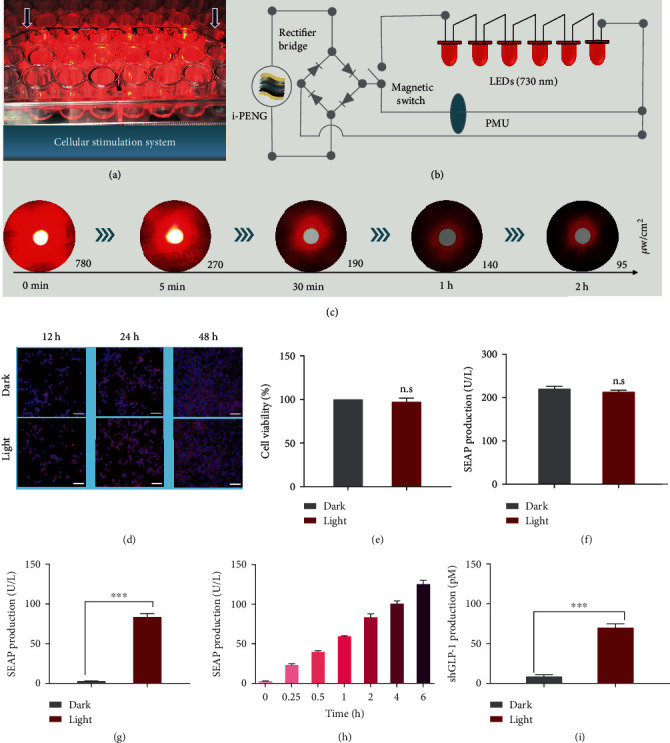
Validating the functionality of SOS-mediated transgene expression in mammalian cells. (a) Photograph and (b) diagram of the cellular stimulation system. (c) Light intensity of LED changing over time. The light intensity was measured with an optical power meter at the indicated time. (d) Immunofluorescence staining images of HEK-293 cells at 12 h/24 h/48 h after illumination. F-actin (red) and DAPI (blue) (scale bar = 100 *μ*m). (e) Viability of cells after FRL illumination. (f) Cell metabolic integrity assay by reporter proteins expression. (g) Activation of the SEAP gene with the SOS. HEK-293 cells transfected with pFR1 and pFR2 were illuminated with FRL LED (730 nm) powered by the PMU. (h) Illumination time–dependent transgene expression with the SOS. (i) Activation of the shGLP-1 gene with the SOS. ^∗∗∗^*P* < 0.001. Data are expressed as means ± SD; *n* = 3 independent experiments. *P* values were calculated by two-tailed unpaired *t*-test. n.s: not significant.

To demonstrate the ability of the i-PENG/PMU to power the LED and ultimately activate optogenetic designer cells to produce therapeutic outputs, we constructed a cellular stimulation system. In this system, engineered cells were cultured in a 96-well plate attached to LEDs being powered by the i-PENG and its associated PMU (Figures 4(a) and 4(b)). To further validate the feasibility of the SOS activating photosensitive engineered cells to produce an output, we measured the illumination intensity of the powered LED over time (Figure 4(c)). The light maximum intensity of a single LED was about 780 *μ*W/cm^2^. Moreover, the light intensity was still maintaining at 95 *μ*W/cm^2^ after 2 h of LED operation, which was strong enough to enable the activation of far-red light-controlled transgene expression [12].

Next, we verified the biocompatibility of using far-red light in the presence of cells. The F-actin cytoskeleton of HEK-293 cells was examined by immunofluorescence assay following light treatment. When observing the total cellular F-actin, we found no difference between cells illuminated with or without far-red light (Figure 4(d)). Moreover, no cytotoxicity on cells was observed under far-red light illumination according to the cell viability assay (Figure 4(e)). As shown in Figure 4(f), we used a metabolic integrity assay to further validate that far-red light had no negative effect on the overall genetic expression capacity of transfected cells. We next assessed the activation kinetics of the SOS-mediated optogenetic transgene expression (Figures 4(g)–4(i)). The data showed that the optogenetic designer cells illuminated with far-red light for 2 h demonstrated a more than 20-fold transgene expression increase compared with dark control cells (Figure 4(g)). SOS-mediated transcriptional activation of optogenetic designer cells exhibited illumination time-dependent kinetics (Figure 4(h)). To explore the therapeutic potential of the SOS, we replaced the reporter protein SEAP with the therapeutic shGLP-1, and the shGLP-1 expression could be induced by far-red light illumination (Figure 4(i)). Taken together, the SOS is biocompatible for further application in optogenetic therapies.

### 2.5. Restoration of Blood Glucose Mediated by the SOS in Diabetic Mice

**Figure 5:**
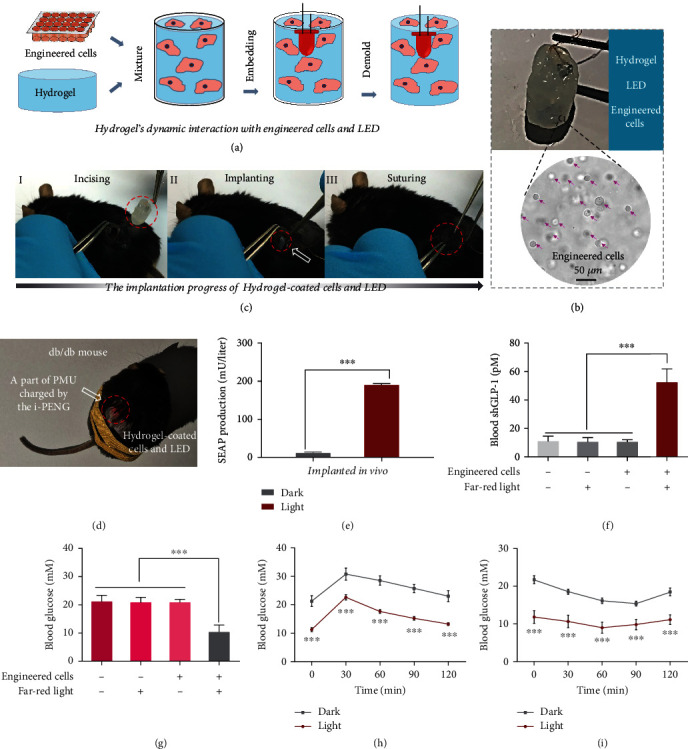
Controlling blood glucose homeostasis using the SOS in a diabetic mouse model. (a) Schematic diagram of hydrogel's dynamic interaction with optogenetically engineered HEK-293 cells and LED. (b) Photograph of the hydrogel-coated optogenetically engineered HEK-293 cells and LED. (c) Implantation progress of hydrogel-coated optogenetically engineered HEK-293 cells and LED. (d) Photograph of the mice after implantation of the SOS. (e) SEAP production of implanted mice exposed to FRL or kept in dark. (f)–(i) The db/db mice with implants were illuminated with FRL for glucose restoration. (f) shGLP-1 production in the treated or nontreated mice. (g) Blood glucose value in the treated or nontreated mice. (h) Glucose tolerance test of mice implanted with the SOS. (i) Insulin tolerance test of mice implanted with the SOS. ^∗∗∗^*P* < 0.001. Data are expressed as mean ± SEM; *n* = 5 mice. *P* values were calculated by two-tailed unpaired *t*-test.

After successfully demonstrating the functionality of the SOS in vitro, we next tested its potential for controlling blood glucose in a diabetic mouse model. To apply the SOS *in vivo*, the engineered optogenetic designer cells and LED were encapsulated within hydrogel, which was shaped in a 48-well plate (Figure 5(a)). The photograph captured by a microscope camera revealed that engineered designer cells were distributed evenly within the hydrogel (Figure 5(b)). Then, the hydrogel was implanted under the dorsum skin of a mouse as indicated (Figure 5(c)). After implantation of the SOS in mice, the LED was powered by the button cell charged by the i-PENG (Figure 5(d)). Therefore, we established a sustainable power supply for the implanted LED which then stimulated designer cells independent of external devices, thus removing behavioral constraints such as external optic fibers or cables (Supplementary Movie 4). To test the functionality of the SOS *in vivo*, wild-type mice were subcutaneously implanted with the SOS, in which the optogenetic designer cells encoding light-driven expression of SEAP reporter were illuminated for 6 h each day. We found that SEAP levels in the bloodstream were significantly increased, indicating that the SOS was providing a sustainable supply of power to facilitate optogenetic stimulation of designer cells *in vivo* (Figure 5(e)). To further validate the therapeutic applications of the SOS, diabetic mice were equipped with the SOS in which the optogenetic designer cells expressed shGLP-1 upon light stimulation (Figure 5(f)). When cells were illuminated for 6 h each day, we found that the amount of shGLP-1 expressed was sufficient to restore homeostatic fasting glycemia (Figure 5(g)), improve glucose tolerance (Figure 5(h) and Figure S5), and insulin sensitivity (Figure 5(i)), as well as reduce insulin resistance (Figure S6). These results suggest that the SOS is sufficiently effective in modulating glucose homeostasis and supports the use of the SOS in long-term optogenetics-based therapy.

## 3. Discussion

Providing sustainable power for light stimulation of optogenetic designer cells is challenging, especially when considering requirements for therapeutic compliance. Hence, it is critical to establish a sustainable power source that satisfies the self-powered and portability needs of implantable optogenetic therapies. For the implantable medical electronics, patients need to go to the hospital periodically to replace the battery, which increases the risk of exogenous infection during surgery and reduces patient compliance. We have developed the SOS as a proof-of-concept to show that rapid restoration of blood glucose homeostasis is possible using the SOS. The SOS was designed to use the i-PENG, which can convert biomechanical energy into electricity for powering an LED that stimulates optogenetic designer cells to produce shGLP-1. We observed that the SOS developed here was sufficiently effective in providing self-powered modulation of blood glucose, supporting its use in powering optogenetic-based cell therapy. Notably, i-PENG can provide long-term sustainable energy by using the biomechanical force of respiration, while also avoiding the limitations associated with external optical fibers, cables, or power suppliers. As a result, the SOS has excellent potential in supporting long-term energy supply for optogenetic-based gene and cell therapies, which is a promising method with high compliance for electronic medicine in the future.

In addition, with the improvement of the efficiency for light-inducible transgene expression of engineered cells, it is possible to directly power LEDs *via* the i-PENG by converting the mechanical energy of respiratory motion into electricity to induce excitation of engineered cells for regulating blood glucose. Note that we recently reported a much more sensitive photoswitch—a red/far-red light-mediated and miniaturized *Δ*phytochrome A- (*Δ*PhyA-) based photoswitch (REDMAP) [46]—which demonstrated that only minutes of illumination can lower blood glucose in mice and rats, decreasing the energy consumption and promising to be applied for the SOS for sustainable implantable blood glucose control in our future work. As a biomechanical energy harvester, the i-PENG has good mechanical properties and robust packaging structure, which has the ability to operate for a long time i*n vivo*. Given the successful use of the SOS for the regulation of blood glucose outlined in this study, it should be relatively straightforward to extend this concept to the treatment of other diseases. In other chronic diseases (e.g. hypertension, gout, obesity), the SOS could be used to encode various therapeutic outputs (e.g., hormones, enzymes, and antibodies). Taking advantage of semiconductor synthetic biology, we can further integrate electronic technology and software development technology into the SOS used in this study, additionally enhancing the SOS to be able to continuously monitor glucose fluctuations in real-time and regulate on-demand release of hypoglycemic agents. We anticipate the SOS concept shown here to be highly translational in its application of supporting long-term optogenetic-based cell therapy.

## 4. Materials and Methods

### 4.1. Fabrication of the SOS

First, the PVDF film with the silver electrodes (60 mm × 20 mm × 110 *μ*m) was attached to the PI substrate (62 mm × 22 mm × 200 *μ*m). Then, the copper tape was employed for fixing wires on the electrodes. The ultrathin PET film (62 mm × 22 mm × 20 *μ*m) was used to package the device. Afterward, the i-PENG was assembled by gluing all parts together via silicone polymers one by one. With the parylene coating system (PDS2010 Labcoter2), parylene-C was coated onto the surface of the device after it had been dried naturally. Finally, the i-PENG and LED were connected to a self-made flexible circuit board integrated with a rectifier bridge, a button cell, and magnetron switch, which were encapsulated by the polydimethylsiloxane (PDMS).

### 4.2. Characterization Methods

The *V*_OC_, *I*_SC_, and *Q*_SC_ were measured with an electrometer (Keithley 6517B) and recorded with an oscilloscope (Teledyne LeCroy HD 4096). The light intensity of the LED was measured by an optical power meter (Q8320 ADCMT).

### 4.3. Cell Viability Assay

Dulbecco's Modified Eagle's Medium (DMEM, Gibco), 1% penicillin/streptomycin (Gibco), and 10% fetal bovine serum (FBS, Gibco) were used to culture the L-929 fibroblasts in a 25 cm^2^ flask, and the cells were incubated at 37°C in 5% CO_2_ atmosphere. Cell Count Kit-8 (CCK-8, Dojindo Molecular Technologies, Inc. Japan) was used to evaluate the proliferation of the cells. Briefly, we cultured L929 cells in a naked 96-well disposable plate coated with parylene-C film on an initial density of 1 × 10^6^ cell/well under the log phase of growth. The cell viability was evaluated every day for 1, 2, and 3 days. Then, 210 *μ*L of complete medium including 10 *μ*L of CCK-8 reagent were added after the culture medium was removed, and the cells were washed by phosphate-buffered saline solution (PBS). After incubation for 1.5 h, 150 *μ*L culture supernatant was transferred into a 96-well plate. A synergy H1 hybrid multimode microplate reader (BioTek Instruments Inc., USA) installed with the Gen5 software (version: 2.04) was employed to measure the absorbance at 450 nm.

### 4.4. Cell Morphologic Observation

The L929 fibroblasts were cultured for 1, 2, and 3 days on the naked 96-well disposable confocal dish and the dish coated with parylene-C film for cell morphologic observation. During staining, the cells were rinsed gently 3 times with phosphate buffer saline, fixed for 10 minutes with 4% paraformaldehyde, and permeabilized for 10 minutes with 0.1% Triton X-100. The fixed cells were then stained with phalloidin-Alexa Fluor 488 (Abcam, Cambridge, MA) for 40 minutes at room temperature and washed 3 times with PBS. Finally, a laser scanning confocal microscope (Leica SP8) was used to observe the stained cells.

### 4.5. Plasmid Construction

All genetic expression vectors are described as follows: pFR1: a constitutive *P*_hCMV_-driven rTA-BldD (a fusion transactivator), BphS (a bacterial photoreceptor), and YhjH (bacterial c-di-GMP phosphodiester) expression vector [*P*_hCMV_-rTA-BldD-pA::P_hCMV_-BphS-P2A-YhjH-pA]; pFR2: a FRL-responsive SEAP expression vector [(whiG)_3_-*P*_hCMVmin_-SEAP-pA]; pFR3: a FRL-responsive shGLP-1 and SEAP expression vector [(whiG)_3_-*P*_hCMVmin_-shGLP-1-P2A-SEAP-pA]; pcDNA3.1(+): a constitutive mammalian *P*_hCMV_-driven expression vector (*P*_hCMV_-MCS-pA) (Invitrogen' CA); and pSEAP2Control: a constitutive *P*_SV40_-driven SEAP expression vector (*P*_SV40_-SEAP-pA) (Clontech' CA). Some expression plasmids were constructed following standard cloning protocols by ClonExpress® MultiS One Step Cloning Kit (Catalog no. C113-01; Vazyme Inc.) and were validated by Sanger sequencing (Genewiz Inc.).

### 4.6. Cell Culture and Transfection

Human embryonic kidney cells (HEK-293, CRL-1573; ATCC) were cultured in DMEM (catalog. no. C11995500BT; Gibco) containing 10% (v/v) FBS (catalog. no. 16000-044; Gibco) and 1% (v/v) penicillin/streptomycin antibiotic solution (catalog. no. ST488-1/ST488-2; Beyotime Inc.). The cells were cultured in a humidified atmosphere at 37°C and 5% CO_2_, and testing for mycoplasma and bacterial contamination was done routinely. Transfection was performed using polyethyleneimine (PEI, molecular weight 40,000; catalog no. 24765; Polysciences) according to an optimized PEI-based protocol [45]. Briefly, 1.2 × 10^4^ cells were plated into a 96-well cell culture plate 18 h before transfection and then cotransfected with pFR1 and pFR2/pFR3 at a ratio of 1 : 1 (w/w) for 6 h with 25 *μ*L PEI (stock solution 1 mg/mL in ddH_2_O) and DNA mixture (PEI and DNA at a mass ratio of 3 : 1). Cell concentration and viability were determined with Countess II (Life Technologies, USA).

### 4.7. SEAP Assay

The quantification of SEAP production in culture supernatant was conducted as previously reported [38]. In brief, the substrate solution was configured with 2 × assay buffer (containing 1 mM MgCl_2,_ 21% (w/w) diethanolamine, 20 mM homoarginine (catalog. no. 1483-01-8, Sangon Biotech, Inc.) (pH 9.8)), and substrate (containing 120 mM p-nitro phenyl phosphate (Catalog. no. 333338-18-4, Sangon Biotech, Inc.)) at a ratio of 5 : 1 (v/v). Then, 120 *μ*L of substrate solution was added to 80 *μ*L heat-inactivated (incubation for 30 min at 65°C) culture medium, and the 405 nm absorption was measured using multimode microplate reader (BioTek Instruments, Inc., USA). Blood SEAP in mice was measured using a chemiluminescence-based assay kit (catalog no. 11779842001, Roche Diagnostics GmbH, Inc.).

### 4.8. Immunofluorescence Staining

HEK-293 cells were cultured on the naked disposable confocal dish and exposed to far-red light (730 nm; 1 mW/cm^2^) for 1 h or placed in the dark. The cells were stained with phalloidin as described above and followed with staining with DAPI for 15 min. The stained cells were visualized by a fluorescence microscope (DMI8; Leica) equipped with a digital camera (DP71; Olympus).

### 4.9. Cell Viability Assay

HEK-293 cells were cultured on the 96-well cell culture plate and exposed to far-red light (730 nm; 1 mW/cm^2^) for 1 h or placed in the dark. Cell Counting Kit-8 (CCK8, Catalog no. C0037; Beyotime, Inc.) was used to evaluate cell viability of the cells at 48 hours after illumination. After incubation with 10 *μ*L of CCK8, the 450 nm absorption was determined using a multimode microplate reader (BioTek Instruments, Inc, USA).

### 4.10. Metabolic Integrity Assay

6 × 10^4^ cells were cultured onto a 24-well cell culture plate and transfected with pSEAP2control. Then, the cells were exposed to far-red light (730 nm; 1 mW/cm^2^) for 1 h or placed in the dark, and SEAP production was measured 48 h after illumination.

### 4.11. SOS Powered Transgene Expression in HEK-293 Cells

Briefly, 1.2 × 10^4^ HEK-293 cells were plated in a 96-well plate and cultivated for 24 hours. The cells were then transfected for 6 h with pFR1 and pFR2 (for SEAP) or pFR3 (for shGLP-1) at a ratio of 1 : 1 (w/w). 18 hours later, the cell culture plate was illuminated with far-red light LED (730 nm; Shenzhen Bestled Opto-electronic Co., Ltd.) powered by the SOS for different time periods. SEAP or shGLP-1 production was measured 48 h after illumination.

### 4.12. Mouse Experiments

HEK-293 cells (2 × 10^6^) transfected with pFR1 and pFR2/pFR3 at a ratio of 1 : 1 (w/w) were suspended in 250 *μ*L 1.5% (w/v) sodium alginate buffer. Subsequently, the cell suspension was transferred into a well of 48-well culture plate, with a LED placed in the middle, solidified for 10 min by adding equal volume of polymerization buffer (containing 10 mM MOPS and 100 mM CaCl_2_ (pH 7.2)). Subsequently, 0.05% poly-L-lysine solution (containing 10 mM MOPS, 0.05% poly-L-lysine, and 0.85% NaCl (pH 7.2)) was added for incubation for another 10 min to obtain the hydrogel/LED implant. For testing the function of SOS in wild-type mice, mice (12-week-old, male, C57BL/6 J, ECNU Laboratory Animal Center) were then implanted with the hydrogel/LED implant containing HEK-293 cells engineered with pFR1 and pFR2, and the engineered cells were illuminated for 2 days (6 h each day; 3 button cells from SOS). Blood samples were collected after blood clotting (37°C for 0.5 h and then 4°C for 2 h) and separation by centrifugation (6000 × g for 10 min) for SEAP assay at 48 h after the first illumination. For db/db mouse treatment, 12-week-old male db/db mice (BKS.Cg-Dock7m +/+ Leprdb/J, derived from C57BL/6 J mice, Charles River Laboratories) were then implanted with the hydrogel/LED implant containing HEK-293 cells transfected with pFR1 and pFR3. Mice were implanted with the hydrogel/LED containing HEK-293 cells transfected with an empty vector pcDNA3.1(+) and were set as negative controls. The engineered cells were illuminated as described above. Forty-eight hours after the first light treatment, blood samples were collected for analytical assays, and physiological tests were performed.

### 4.13. Rat Experiments

Sprague Dawley (SD) rats (250-300 g, male, Sprague Dawley, Beijing Vital River Laboratory Animal Technology Co. Ltd) were used for biomechanical energy harvesting *via* i-PENG *in vivo*. The anesthesia procedure for rats started with inhalation of isoflurane gas (1-3% in pure medial grade oxygen), followed by intraperitoneal injection of 1% sodium pentobarbital (40 mg kg^−1^) for anesthesia induction and maintenance, respectively. After anesthesia, the chest skin was incised for implanting the i-PENG.

### 4.14. Intraperitoneal Glucose Tolerance Test (IGTT)

After fasting for 16 h, the mice were intraperitoneally injected with aqueous D-glucose (1 g/kg, Sangon Biotech; Cat. no. G0188, Lot. no. AA09BA0019). The glycemic profile was monitored by Contour Glucometer (Bayer) via tail-vein blood samples at indicated timepoints after glucose administration. The area under the curve (AUC) for GTT was determined by the trapezoidal rule.

### 4.15. Insulin Tolerance Test (ITT)

After fasting for 4 h, the mice were then intraperitoneally injected with recombinant human insulin (1 U/kg, Cat. No. I3536, Sigma-Aldrich). The glycemic profile was monitored at indicated timepoints after insulin administration.

### 4.16. Homeostasis Model Assessment of Insulin Resistance (HOMA-IR)

The resistance index of insulin resistance was calculated using the HOMA-IR equation: HOMA − IR = glucose (mmol/L) × insulin (mU/L)/22.5.

### 4.17. ELISA

Quantification of the shGLP-1 expression in culture supernatant and mouse serum was facilitated using a high-sensitivity GLP-1 Active ELISA kit (Cat. no. EGLP-35 K, Lot. no. 2639195; Merck Millipore Corporation). The insulin expression in mouse serum was measured using a mouse insulin ELISA Kit (Cat. no. 10-1247-01, Lot. no. 24243; Mercodia AB).

### 4.18. Ethics

The experiments involving animals were performed according to the protocol (protocol ID: R + m 20210601) approved by the ECNU Animal Care and Use Committee and in direct accordance with the Ministry of Science and Technology of the People's Republic of China on Animal Care guidelines. After the termination of the experiments, all animals were euthanized.

### 4.19. Statistical Analysis

All in vitro data are representative of three independent experiments (mean ± SD, *n* = 3). For the animal experiments, each group consisted of randomly selected mice (mean ± SEM, *n* = 5 mice). Student's *t*-test was used for statistical significance analysis. No data were excluded from the study. *P* < 0.01 (^∗∗^) was considered very significant, and *P* < 0.001 (^∗∗∗^) was considered extremely significant. All data was analyzed using GraphPad Prism software version 6.0. *n* and *P* values are presented in the figure legends.

## Data Availability

All data needed to evaluate the conclusions in the paper are present in the paper and/or the Supplementary Materials. Additional data related to this paper may be requested from the authors.

## Supplementary Materials

Supplementary Materials Figure S1: SEM images of the i-PENG. Figure S2: flexibility test of the i-PENG. Figure S3: demonstration of the i-PENG for mechanical energy harvesting. Figure S4: fatigue performance data of the i-PENG. Figure S5: area under the curve (AUC) analysis of the IGTT data from Figure 5h. Figure S6: insulin resistance (HOMA-IR) analysis of mice implanted with the SOS. Movie S1: a LED lighted by the i-PENG in vivo. Movie S2: the SOS controlled by a magnetic switch in vitro. Movie S3: the SOS controlled by a magnetic switch in vivo. Movie S4: a db/db mouse after implanted the SOS.
