# Supplementary material for: A Self-Powered Optogenetic System for Implantable Blood Glucose Control
Source: Research (Wash D C). 2022 Jun 16;2022:9864734. doi: 10.34133/2022/9864734 (PMC9275083; doi:10.34133/2022/9864734)
Supplement: Supplementary Materials — Figure S1: SEM images of the i-PENG. Figure S2: flexibility test of the i-PENG. Figure S3: demonstration of the i-PENG for mechanical energy harvesting. Figure S4: fatigue performance data of the i-PENG. Figure S5: area under the curve (AUC) analysis of the IGTT data from Figure 5h. Figure S6: insulin resistance (HOMA-IR) analysis of mice implanted with the SOS. Movie S1: a LED lighted by the i-PENG in vivo. Movie S2: the SOS controlled by a magnetic switch in vitro. Movie S3: the SOS controlled by a magnetic switch in vivo. Movie S4: a db/db mouse after implanted the SOS. [file 9864734.f1.zip › Revised Supporting information publication.docx]

A Self-powered Optogenetic System for Implantable Blood Glucose Control

Zhuo Liu^1,2^, Yang Zhou^3^, Xuecheng Qu^2^, Lingling Xu^2^, Yang Zou^2^, Yizhu Shan^2^, Jiawei Shao^3^, Chan Wang^2^, Ying Liu^2^, Jiangtao Xue^2^, Dongjie Jiang^2^, Yubo Fan^1*^, Zhou Li^2,4,5,6*^, Haifeng Ye^3,7*^

1. Key Laboratory for Biomechanics and Mechanobiology of Ministry of Education, Beijing Advanced Innovation Centre for Biomedical Engineering, School of Biological Science and Medical Engineering, School of Engineering Medicine Beihang University, Beijing 100191, China

2. CAS Center for Excellence in Nanoscience Beijing Key Laboratory of Micro-nano Energy and Sensor, Beijing Institute of Nanoenergy and Nanosystems, Chinese Academy of Sciences, Beijing 101400, China

3. Shanghai Frontiers Science Center of Genome Editing and Cell Therapy, Biomedical Synthetic Biology Research Center, Shanghai Key Laboratory of Regulatory Biology, Institute of Biomedical Sciences and School of Life Sciences, East China Normal University, Dongchuan Road 500, Shanghai 200241, China

4. School of Nanoscience and Technology, University of Chinese Academy of Sciences, Beijing 100049, China

5. Center of Nanoenergy Research, School of Physical Science and Technology, Guangxi University, Nanning 530004, China

6. Institute for Stem Cell and Regeneration, Chinese Academy of Sciences, Beijing 100101, China

7. Chongqing Key Laboratory of Precision Optics, Chongqing Institute of East China Normal University, Chongqing 401120, China.

Correspondence should be addressed to Yubo Fan; [yubofan@buaa.edu.cn](mailto:yubofan@buaa.edu.cn) and Zhou Li; [zli@binn.cas.cn](mailto:zli@binn.cas.cn) and Haifeng Ye; [hfye@bio.ecnu.edu.cn](mailto:hfye@bio.ecnu.edu.cn)

Figure S1: SEM images of the i-PENG. (a) SEM images of surface structure of the PVDF film. (b) SEM images of surface structure of the PVDF film coated with Ag electrodes. (c) SEM image of cross-sectional of the i-PENG.


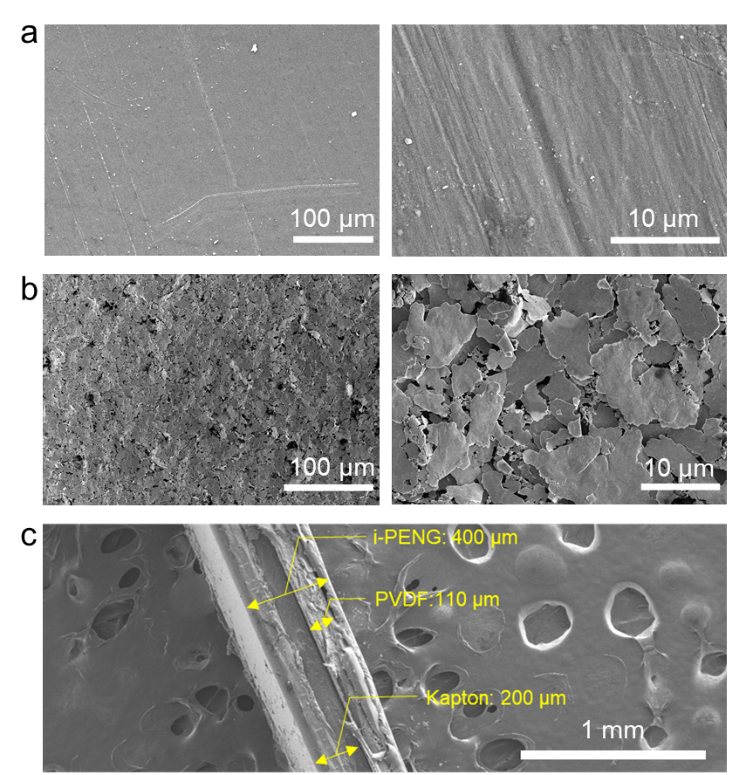

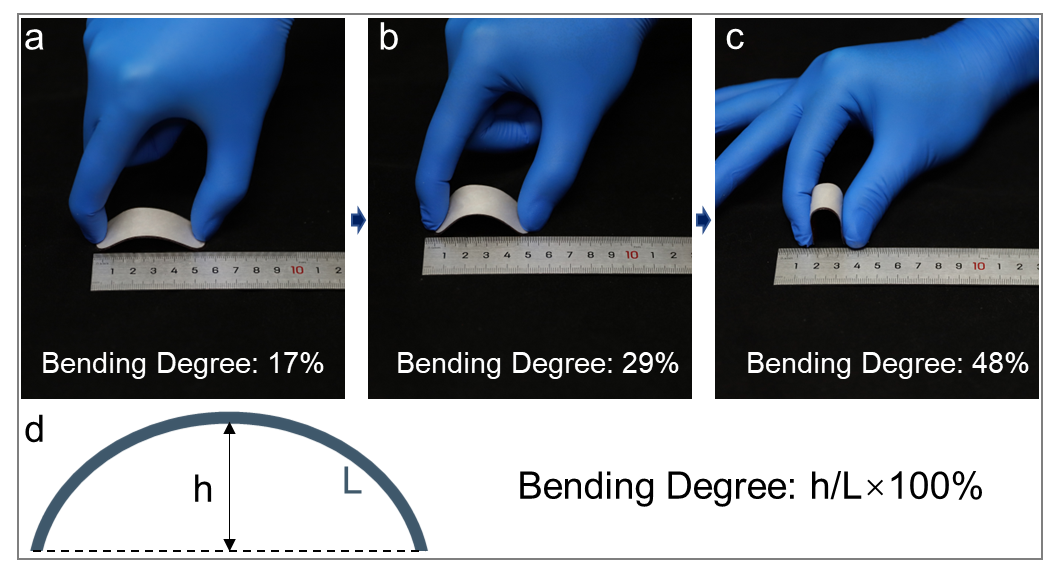


Figure S2: Flexibility test of the i-PENG. (a-c) Different bending states of the i-PENG and their relative bending degree. (d) Schematic graph of the calculation method.


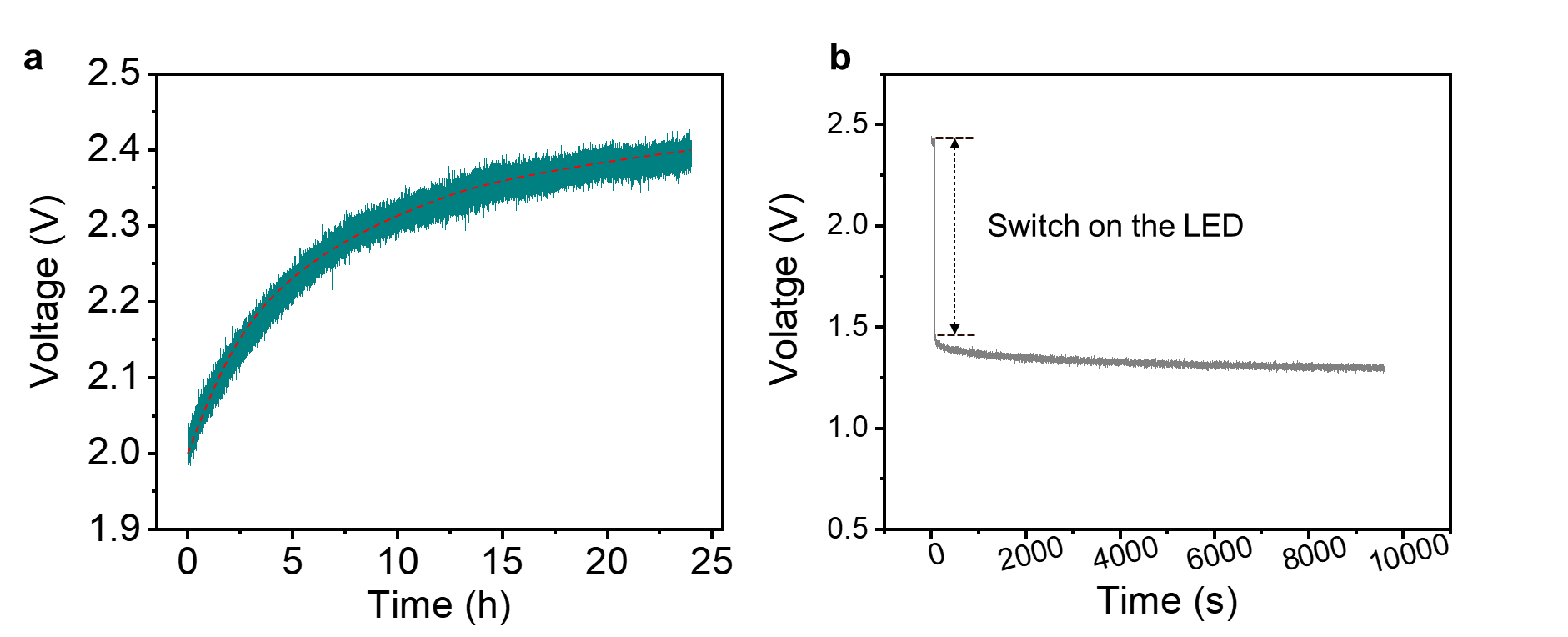


Figure S3: Demonstration of the i-PENG for mechanical energy harvesting. (a) Charging and (b) discharging curve of a button cell by the i-PENG and LED, respectively.


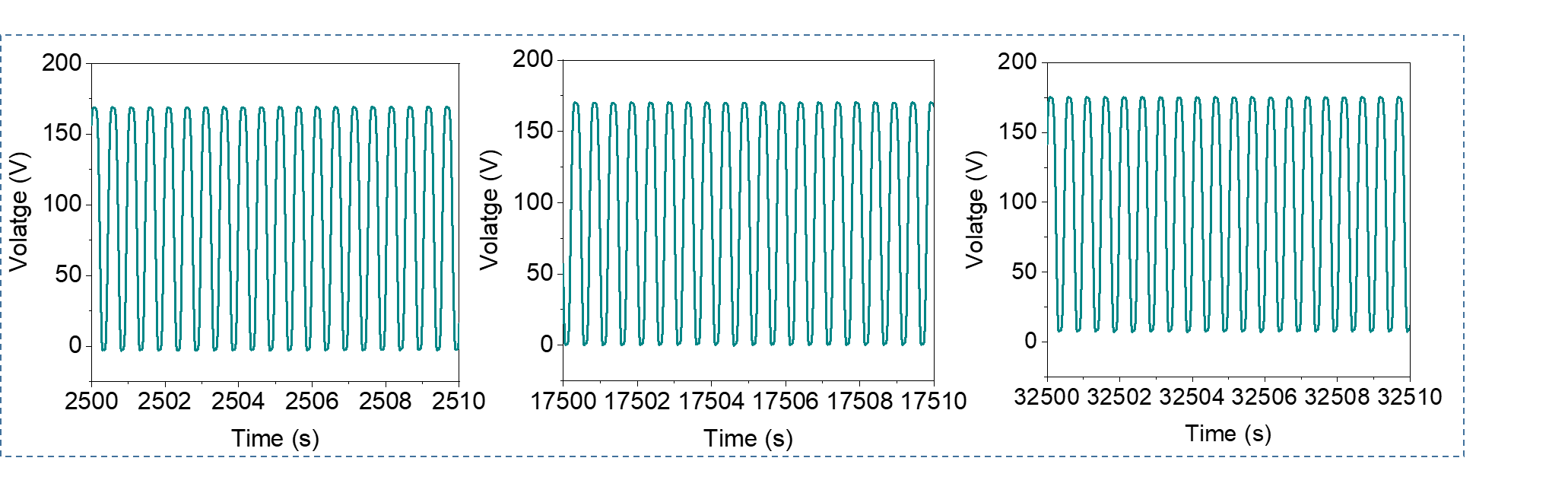


Figure S4: The voltage details within 10 s at the beginning, in the middle part and at the end of fatigue test.

Figure S5: Area under the curve (AUC) analysis of the IGTT data from Figure 5h.

Figure S6: Insulin resistance (HOMA-IR) analysis of mice implanted with the SOS.
